# Impact of fluid resuscitation volume on the severity of organ failures in severely burned patients

**DOI:** 10.1186/cc11068

**Published:** 2012-03-20

**Authors:** N Depaye, G Minguet, A Magnette, D Jacquemin, D Ledoux, P Damas

**Affiliations:** 1 University Hospital of Liege, Belgium

## Introduction

Adequacy of fluid resuscitation remains a cornerstone of early burn management. The Parkland formula - that is, administration of 4 ml/kg/% total of the body surface area (TBSA) burned with Ringer's lactate for the first 24 hours post injury - has been used for decades. The purpose of this study was to evaluate the effect of adherence with the Parkland protocol and its impact on the severity of organ failure during the first week post injury using the Sequential Organ Failure Assessment (SOFA) score.

## Methods

We conducted a retrospective review of burns' resuscitation data, from 2000 to 2007, on 101 adult patients (aged ≥16 years) admitted within the first 24 hours following injury, with a %TBSA burned of 20 or more. A classification of patients into four groups, according to fluids administered, was done for comparison between these groups. The SOFA score was calculated daily for the first week after admission. The neurological component of SOFA was left out because of the difficulty to assess the actual Glasgow Coma Scale in sedated patients. Organ failures were defined by partial SOFA ≥3. Data are expressed as median (Q1 to Q3) and are analyzed using the chisquare test (*P *< 0.05 was considered statistically significant).

## Results

A total of 62 patients with complete data on fluid administration were included in the analysis. Median age was 41 (28 to 54) years, median TBSA burned was 35.5 (25 to 50); median ICU stay was 38 (12 to 62) days and 13 (21%) patients died. Ten patients suffering from inhalation injury were excluded from further analysis. Median fluids administered was 4.9 (4.1 to 6.2) ml/kg/%TBSA at 24 hours. Five patients received <3. 5ml/kg/%TBSA, 15 between 3.5 and 4.5 ml/kg/%TBSA, 17 between 4.5 and 6 ml/kg/%TBSA and 15 patients >6 ml/kg/%TBSA. No differences existed between groups concerning the cause and surface of injury, age, sex, and comorbidities. Compared to others, patients who received >6 ml/kg/%TBSA had a significant increase in respiratory failure (*P *= 0.03). The amount of fluids administered had no impact on the incidence of cardiovascular (*P *= 0.89), renal (*P *= 0.11), liver (*P *= 0.52) and coagulation (*P *= 0.86) failure.

## Conclusion

This single-centre retrospective study indicates that fluid resuscitation volumes frequently overcome those previously established by the Parkland protocol. This fluid over-resuscitation may have deleterious effects on patient outcome by increasing the incidence of respiratory failure.
